# Neural Basis of Limb Ownership in Individuals with Body Integrity Identity Disorder

**DOI:** 10.1371/journal.pone.0072212

**Published:** 2013-08-21

**Authors:** Milenna T. van Dijk, Guido A. van Wingen, Anouk van Lammeren, Rianne M. Blom, Bart P. de Kwaasteniet, H. Steven Scholte, Damiaan Denys

**Affiliations:** 1 Department of Psychiatry, Academic Medical Center, University of Amsterdam, Amsterdam, The Netherlands; 2 Brain Imaging Center, Academic Medical Center, University of Amsterdam, Amsterdam, The Netherlands; 3 Department of Psychology, University of Amsterdam, Amsterdam, The Netherlands; 4 Netherlands Institute for Neuroscience, an institute of the Royal Netherlands Academy of Arts and Sciences, Amsterdam, The Netherlands; 5 Sackler Institute of Graduate Biomedical Sciences, New York University School of Medicine, New York, New York, United States of America; Royal Holloway, University of London, United Kingdom

## Abstract

Our body feels like it is ours. However, individuals with body integrity identity disorder (BIID) lack this feeling of ownership for distinct limbs and desire amputation of perfectly healthy body parts. This extremely rare condition provides us with an opportunity to study the neural basis underlying the feeling of limb ownership, since these individuals have a feeling of disownership for a limb in the absence of apparent brain damage. Here we directly compared brain activation between limbs that do and do not feel as part of the body using functional MRI during separate tactile stimulation and motor execution experiments. In comparison to matched controls, individuals with BIID showed heightened responsivity of a large somatosensory network including the parietal cortex and right insula during tactile stimulation, regardless of whether the stimulated leg felt owned or alienated. Importantly, activity in the ventral premotor cortex depended on the feeling of ownership and was reduced during stimulation of the alienated compared to the owned leg. In contrast, no significant differences between groups were observed during the performance of motor actions. These results suggest that altered somatosensory processing in the premotor cortex is associated with the feeling of disownership in BIID, which may be related to altered integration of somatosensory and proprioceptive information.

## Introduction

The feeling that our body belongs to us (‘body ownership’) appears so self-evident that it is difficult to believe otherwise. Remarkably, there are individuals that do not have the experience that all of their limbs belong to them. Individuals with the extremely rare condition body integrity identity disorder (BIID; also known as apotemnophelia and xenomelia) describe a feeling of alienation and ‘overcompleteness’ for a particular limb, and wish for amputation of perfectly healthy body part(s). However, they do not suffer from delusions or psychosis and fully understand the bizarre nature of their wish. BIID is associated with significant functional impairment, but there is currently no effective pharmacological or psychotherapeutic treatment option. Interestingly, individuals that have undergone surgery consider amputation helpful and report lower disability [1], [2].

The feeling of body ownership has been suggested to be mediated by a frontoparietal network, including multisensory integration areas such as the parietal cortex and premotor cortex, as well as the insula [3]–[5]. Initial lesion studies have observed that patients with parietal lobe damage may develop somatoparaphrenia, a condition in which patients deny the ownership of a limb [6]–[8]. A more recent lesion mapping study suggested that the insula is most often affected in patients with disturbed sensation of limb ownership [9]. Also damage to the premotor cortex has been associated with the lack of awareness of a limb (asomatognosia) in a case report [10]. Other studies have investigated the feeling of ownership by making use of perceptual illusions. For example, by synchronous stroking of a rubber hand and one’s own unseen hand, participants tend to feel that the rubber hand is theirs [11]. Neuroimaging studies of this rubber hand illusion in healthy individuals have implicated the ventral premotor cortex (PMv) in the feeling of body ownership [12], [13]. However, research with stroke patients showed that although intact white matter connections to the PMv are necessary for the body ownership illusion, patients with asomatognosia did not seem to have damage in regions connected to the PMv [14]. This has led to the suggestion that an intact PMv is necessary to experience the rubber hand illusion, rather than mediating the actual feeling of body ownership [15]. In addition, a blood flow positron emission tomography (PET) study in healthy individuals found that maintaining a limb ownership illusion is associated with increased activation in the right posterior insula [16]. Thus, the different approaches that have been used to investigate body ownership do support the involvement of the frontoparietal network and insula but point to other nodes of the network mediating the feeling of ownership.

Various neural models for BIID have been proposed on the basis of these and other observations [17], [18], but only recently studies have started to investigate the neural mechanisms of limb ownership in BIID. Individuals with BIID were reported to have altered cortical thickness and surface area in the parietal cortex and insula [19]. Another study reported reduced parietal cortex responses to tactile stimulation of the affected limb [20]. These studies focused on the parietal cortex and insula but did not report about the possible involvement of the PMv. In the present study, we investigated neural responses across the whole brain to tactile stimulation of the leg that felt alienated, as well as the other leg that felt as a normal part of their body while recording blood oxygen level dependent (BOLD) functional magnetic resonance imaging (MRI). We included a group of matched controls to account for normal responsivity to tactile stimulation. The participants also performed a motor execution task to determine whether the feeling of body ownership primarily originates in the somatosensory network or whether the motor network is involved.

## Materials and Methods

### Participants

Five male individuals with BIID and ten male matched healthy control participants (age mean ± SD, BIID: 36.6±10.3, controls: 42.0±11.6, t(13) = 0.88, p = 0.39; level of education (ISCED): BIID: 4.6±0.9, controls: 4.5±0.8, t(13) = −0.21, p = 0.84; handedness (right/left): BIID: 4/1, controls: 9/1, χ^2^(1) = 0.29, p = 0.59) were recruited through advertisements on online forums and via other participants. BIID was diagnosed with a psychiatric assessment, using the definition of BIID as the existence of a lifelong desire to have an amputation with the primary objective to restore one’s true identity [1]. Furthermore, they were screened for the presence of DSM axis I and II disorders. Characteristics of subjects with BIID are described in Table 1. In summary, one of the BIID participants had comorbid depression. Depression and anxiety have been reported as comorbid symptoms in 21–27.7% of cases [1], [2]. Since the wish for amputation is mostly a lifelong persistent desire and antedates the occurrence of comorbid psychiatric symptoms, this depression is more likely the consequence of suffering due to BIID than that BIID is the consequence of depression. Three individuals with BIID desired amputation of the right leg and two desired amputation of the left leg. Control participants did not have a history of psychiatric disorders. The study was approved by the Medical Ethical committee of the Academic Medical Centre (AMC) of the University of Amsterdam and after complete description of the study to the subjects, written informed consent was obtained.

**Table 1:** Subject characteristics of the individuals with Body Integrity Identity Disorder (BIID).

Subject	Age (years)	Educational level	Handedness	Desired amputation	Onset BIID	Psychiatric comorbidity
1	43	Higher education	R	L upper leg	Childhood	None
2	44	Secondary school	R	R lower leg	Childhood	Depression
3	33	University degree	L	R upper leg; R index finger	Childhood	None
4	20	Secondary school	R	R upper leg	Childhood	None
5	43	University degree	R	L upper leg	Childhood	None

### Procedure and Experimental Paradigms

First, two patches of skin for stroking of ∼4×3 cm^2^ were marked per leg, one on each upper and lower leg above and below the line of desired amputation. The patches on the upper legs were marked 14–18 cm above the knee and 5 cm lateral, the patches on the lower legs were marked 18–22 cm below the knee and 5 cm medial. We chose these configurations because the majority of individuals with BIID reported that they desired amputation just above or below the knee [2]. Thereafter the tasks were explained and practiced.

The tactile stimulation paradigm consisted of thirty 16 second blocks of stimulation alternated with 16 seconds of rest. During rest participants were instructed to relax. The tactile stimulation comprised stroking of one skin patch by use of a brush with a speed of approximately 1 Hz [21]–[23]. Every patch was stimulated during six blocks and the order of the locations was pseudo-randomized (no consecutive blocks on the same patch, to minimize habituation). In addition, there were six blocks in which the experimenter stroked both legs proximal to distal at the same time using two brushes in about 4 seconds, resulting in four whole leg strokes in a 16-second block. We included this latter condition because the first BIID participant noted that simultaneous stroking of his entire legs resulted in “an explosion in his head”. However, none of the other participants noticed a similar effect and we therefore did not include this condition in the final analysis. To help the experimenter keep a steady brushing movement, the 1 Hz rhythm was projected on a screen. The mirror was removed from the head coil to prevent the possibility to see the tactile stimulation, such that the neural activations were unlikely mediated by the integration of visual and tactile information. One BIID participant reported that tactile stimulation of the upper alienated leg felt like it was in the desired amputation area, instead of above or below it. Therefore we decided not to use stimulation of the upper legs as a control condition or the proximal to distal condition and instead only compared stimulation of the lower owned and lower alienated legs.

The motor task consisted of twenty-four 16 second blocks of movement of the digits of one foot or hand alternated with 16 seconds of rest. Participants were cued to flex and point the toes of the left or right foot or to clench and unclench the left or right hand with a speed of 1 Hz [23], [24]. Digits of one limb were moved during 6 blocks and blocks were alternated between limbs in a pseudo-randomized order (no digits of one limb were moved in consecutive blocks). Participants were shown the 1 Hz rhythm on a screen so they knew the speed with which they should perform the movement. One BIID participant had a wish for amputation of his index finger in addition to his leg. We therefore decided to only compare between wiggling of the owned and alienated toes in the motor task.

### Data Acquisition

Magnetic Resonance Imaging was performed with a Philips 3 Tesla Intera MR system. For functional imaging, T2* weighted echo-planar images (EPI) with blood oxygen level dependent (BOLD) contrast were measured while subjects performed the motor and sensory task. Parameter settings were the following: Matrix size: 96×96; FOV: 220×220×122 mm; voxel size: 2.29×2.29×3 mm^3^; slice gap: 0.3 mm; acquisition direction: ascending; TR: 2120 msec.; TE: 30 msec; Flip angle: 80°; no. slices per volume: 40 slices. A structural T1-weighted image was also acquired for spatial normalization purposes, with the following parameters: Matrix size: 256×256, field of view (FOV): 226×226×218 mm^3^, voxel size: 0.88×0.88×1.2 mm^3^, repetition time (TR): 9.6 sec., echo time (TE): 4.6 msec.; Flip angle: 8°; no. slices: 182.

### Data Analysis

MRI data were analysed using FSL (http://fsl.fmrib.ox.ac.uk/fsl/fslwiki/) [25]. Brains were extracted using BET. The functional images were slice time aligned (ascending), motion corrected, highpass filtered (64 sec.), smoothed temporally (FWHM of 2.4 sec.) and spatially (FWHM 5 mm) with a Gaussian filter. Functional images were aligned to the T1-weighted image acquired at the start of the session using normal search with 7 degrees of freedom and transformed, nonlinearly, based of this structural image, to MNI space. Explanatory variables were constructed for each of the tactile and motor conditions. Subsequently, time-series statistical analysis was performed using FILM for local autocorrelation correction. For the tactile stimulation experiment, the contrasts left lower leg>rest and right lower leg>rest were obtained. For the motor execution experiment, the contrasts left toe>rest and right toe>rest were obtained. Higher-level analysis was executed using a mixed effects model (FLAME 1+2) corrected for multiple comparisons across the entire brain using cluster-based thresholding (Z>2.3, p<0.05). In the higher-level analysis the appropriate first-level contrasts were pooled in a control group and a BIID group. To compare neural responses to stroking or moving the owned versus alienated leg in the BIID group, for three individuals with BIID the right leg was subtracted from the left leg, while for the remaining two individuals with BIID the left leg was subtracted from the right leg. This procedure ensures that the higher order neural representations for the alienated leg are aligned across individuals, but disregards potential differences that are specific to the affected side of the body. To control for unequal stroking and moving the right versus left leg, for six control participants the right leg was subtracted from the left leg, while for the remaining four controls the opposite was done to ensure equal left versus right stroking and moving between groups.

## Results

### Somatosensory Stimulation

**Figure 1:**
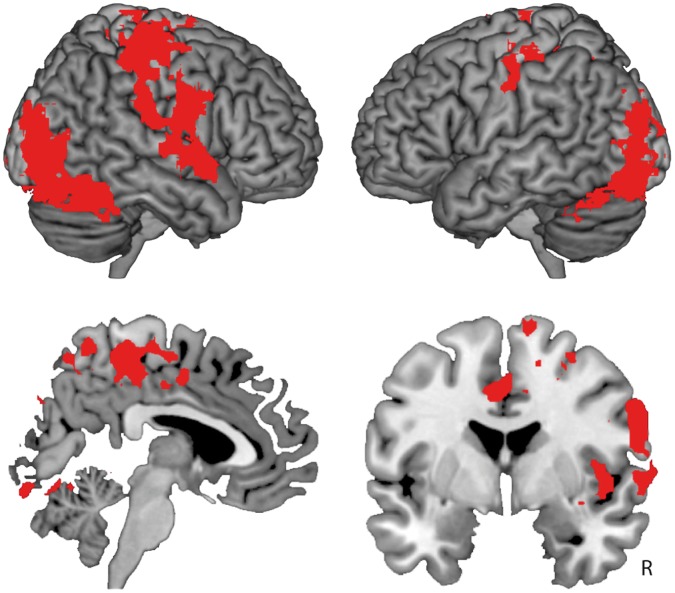
Increased neural responsivity to tactile stimulation in individuals with Body Integrity Identity Disorder (BIID) compared to healthy controls (group main effect). The figure shows two significant clusters in the frontoparietal network and occipitotemporal cortex for the main effect of group. This analysis assesses neural responsivity to tactile stimulation of both legs, irrespective of which leg the individuals with BIID felt as alienated or as a normal part of the body (z>2.3, p<0.05, corrected). The top panels show increased activation in the ventral (PMv) and dorsal (PMd) premotor cortex, the precentral and postcentral gyri, the superior parietal lobule, and the supramarginal gyrus. The bottom left panel shows increased activation in the precuneus, and the bottom right panel the increased activation in the right insula.

We initially assessed whether the responsivity of the somatosensory network was altered in individuals with BIID. Therefore, we compared neural activity during stimulation of the left and right lower legs between the BIID and control groups, irrespective of which leg the individuals with BIID felt as not belonging to their body (i.e., the main effect of group; BIID (left+right>rest) vs. control (left+right>rest)). This analysis showed increased neural responsivity in two large clusters covering the frontoparietal network (main group effect; p<0.001, corrected) and the occipitotemporal cortex (p<0.001, corrected). The frontoparietal cluster included the dorsal premotor cortex (PMd), the precentral and postcentral gyri including the somatosensory cortex, and the superior parietal lobule bilaterally. Furthermore it also included the right PMv, insular cortex and supramarginal gyrus. The cluster in the occipitotemporal cortex included the lateral occipital cortex, the precuneus, inferior temporal cortex, fusiform gyrus and cerebellum bilaterally (see Fig. 1 and Table 2). There were no brain regions that showed significantly reduced responsivity to stimulation in the BIID group. Thus, the frontoparietal network implicated in body ownership was more sensitive to tactile stimulation in individuals with BIID than in controls.

**Table 2:** Local maxima (thresholded at z>3.1) within the significant clusters (z>2.3, p<0.05, corrected) for the main effect of group and the group×ownership interaction.

Region	MNI coordinates			Peak Z
	x	y	z	
*Main effect (BIID>controls)* a				
R inferior occipital cortex	50	−86	−8	5.6
L fusiform gyrus	−16	−84	−16	4.9
R precentral gyrus	24	−24	68	5.1
L precentral gyrus	−12	−32	62	5.1
R precentral gyrus	2	−30	50	4.4
R ventral premotor cortex (PMv)	62	0	34	5.0
R ventral premotor cortex (PMd)	28	−6	56	3.3
R insula	48	10	−8	4.2
R supramarginal gyrus	68	−22	18	4.6
L fusiform gyrus	−22	−52	−18	4.2
L precuneus	−8	−58	54	4.2
L anterior cingulate cortex	−8	0	42	4.4
R supplementary motor area	12	−8	64	3.9
R postcentral gyrus	8	−36	78	3.9
R precuneus	2	−56	64	4.1
L inferior temporal gyrus	−54	−66	−24	3.9
L occipital pole	−12	−88	26	4.2
*Group×ownership interaction*				
L ventral premotor cortex (PMv)	−52	12	24	3.6
L dorsal premotor cortex (PMd)	−58	−10	42	3.2

a Local maxima for clusters with >20 voxels are reported.

**Figure 2:**
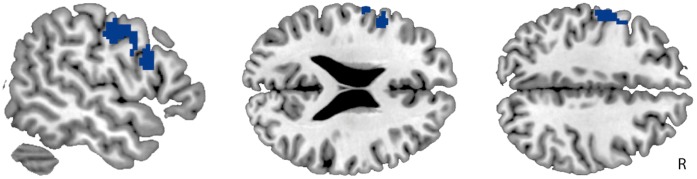
Reduced neural responsivity in the left premotor cortex to tactile stimulation of the leg that felt alienated in individuals with Body Integrity Identity Disorder (BIID; group×ownership interaction). The figure shows one significant cluster including the ventral (PMv; middle) and dorsal (PMd; right). This analysis assesses neural responsivity to tactile stimulation of the leg that felt alienated versus the leg that felt a normal part of the body in individuals with BIID, in comparison to stimulation of the corresponding legs in healthy controls (z>2.3, p<0.05, corrected).

To elucidate the neural activity associated with the absent feeling of body ownership, we compared neural activity during stimulation of the lower leg that felt alienated versus the lower leg that felt a normal part of the body, irrespective of whether this was the left or right leg (i.e., group×ownership interaction; BIID (alienated>owned) vs. control (corresponding left vs. right legs)). Stimulation of the alienated compared to the owned leg in BIID showed reduced activation in a cluster in the contralateral PMv and PMd in comparison to stimulation of the corresponding legs in healthy controls (p<0.007, corrected; see Fig. 2 and Table 2). No other brain regions showed this interaction, not even at a very lenient threshold (p>0.01, uncorrected).

There were no brain regions that showed significantly increased activity during stimulation of the alienated compared to owned leg in the BIID group. Thus individuals with BIID have reduced activation in the PMv and PMd contralateral to their alienated leg during stimulation of that leg.

### Motor Execution

To assess whether the feeling of body disownership was associated with altered activity in the motor network in individuals with BIID, we analyzed the data that was acquired during wiggling of the toes of the leg that felt alienated and the leg that felt a normal part of the body. No significant differences in motor execution activity were observed between the BIID and control group (p>0.05, corrected), not when the feeling of leg ownership was taken into account (group×ownership interaction), nor when the feeling of ownership was not considered (main group effect). This suggests that the feeling of limb disownership in individuals with BIID is not strongly related to altered activity in the motor system.

## Discussion

We investigated the neural basis underlying the feeling of body disownership in individuals with BIID. The results showed heightened sensitivity of the somatosensory network to tactile stimulation in individuals with BIID compared to controls. Importantly, the results also suggest that hypofunction in the PM is associated with the feeling of disownership of alienated limbs, indicating that the PM is involved in body ownership.

A large network including the PM, parietal cortex and insula showed heightened responsivity to stimulation in individuals with BIID, regardless of which leg was stimulated. We speculate that the individuals with BIID may have paid more attention to the tactile stimulation, resulting in increased somatosensory feedback and neural activity [26]. The parietal cortex and insula have been implicated previously in body ownership in neuroimaging and lesion studies [9], [16], [27]. While those studies have suggested that these multisensory integration areas might be necessary for body ownership, our results implicate that these regions do not likely mediate the actual feeling of body ownership, since we did not observe any differences in these areas between stimulation of the leg that felt alienated versus the leg that felt like a normal part of the body. This may explain why some stroke patients with insula lesions do not manifest symptoms of disturbed body ownership [9]. Activity in the insula and parietal cortex may therefore reflect multisensory information processing that is necessary for illusory ownership to occur, but these regions do not appear to contribute to the actual feeling of ownership.

The only brain region that was differentially activated for the owned and alienated leg was the PM. The results of our study therefore provide support for neural models that have suggested that the PM is crucial for body ownership [3]. Previous support for this model has primarily come from studies that used perceptual illusions [12], [13]. Our results now suggest that the PM is also involved in the feeling of ownership for real limbs. The PM has been shown to respond to information from various modalities and is thought to integrate multisensory information [3], [12], [28], [29]. This suggests that the reduced PM BOLD response in BIID may be caused by altered integration of somatosensory and proprioceptive information. The feeling of body ownership is a complex function that involves many different brain regions including the parietal cortex and insula [3], [5]. The PM is structurally and functionally well connected to those brain regions, and may therefore be particularly well situated to integrate information that is distributed across a large brain network [30], [31]. Nevertheless, it remains to be elucidated how altered PM processing eventually leads to the desire to amputate a limb.

In contrast to tactile stimulation, the results showed no significant differences between groups during motor execution. Based on the available models for body ownership we expected that the feeling of limb disownership in BIID would primarily be related to altered representations in the somatosensory network [3]–[5]. To investigate whether the motor network would also be involved, we included a motor execution experiment. Although negative results do not provide evidence for the absence of effects and the analyses are limited in power, these results do suggest that there are at least no large differences in the neural representation of the motor system in BIID.

Recently, two neuroimaging studies in BIID have been published. A structural MRI study reported subtle differences in cortical thickness and surface area across the parietal cortex and insula but did not analyze other brain regions. This suggests that altered PM processing may result from reduced integration of parietal cortex and insula information. However, the results of that study did not survive a stringent correction for multiple comparisons and therefore await replication [19]. Furthermore, a magnetoencephalography (MEG) study compared neural responses in the parietal cortex to tactile stimulation of alienated limbs in participants with BIID to stimulation of owned limbs in BIID and healthy control participants and found decreased right superior parietal lobule activation at 40–140 ms after tactile stimulation [20]. The present results showed that tactile stimulation of the lower legs, whether they are owned or alienated, led to increased activation in the parietal cortex compared to controls during 16 seconds of stimulation, and that the only differential activity distinguishing ownership from disownership lies in the PM. A possible explanation is that BIID is associated with initial parietal hyporesponsivity that is followed by sensitization. This might also account for their increased sympathetic responses to pinprick stimulation [32] and may be mediated by recurrent feedback mechanisms. But although the parietal cortex is involved in body representations, our results and those of a previous transcranial magnetic stimulation (TMS) study suggest that it does not play a causal role in the feeling of body ownership [33]. Additional studies are warranted to reconcile these observations.

These findings suggest that BIID has a neural basis that needs to be recognized as such in order for proper treatment to be developed. Individuals with BIID are often thought to be delusional or attention-seeking, but our and other findings [20] indicate that the dysfunction lies in the body ownership network, congruent with the description of disownership of their alienated limb. Attempts to treat this condition with psychotherapy or pharmacotherapy have been unsuccessful [1], [2], [34]. Recognition of BIID as a condition that is characterized by neural deficits in the body ownership network may lead to the development of new treatment options and provide a rationale for the establishment of a treatment plan. Furthermore, future studies could investigate whether direct modulation of PM activity by endogenous (e.g., neurofeedback using fMRI or electroencephalography (EEG)) or exogenous (e.g., neurostimulation using TMS or transcranial direct current stimulation (tDCS)) methods is effective. This may circumvent surgical procedures that currently appear the only effective treatment.

The main strength of our study is that it allowed to investigate the feeling of ownership of one’s actual limbs, rather than investigating illusory feelings of ownership. A limitation of our study is the small sample size, which may nevertheless be considered reasonable because BIID is an extremely rare condition. The small sample size also required us to disregard whether the left or right leg was affected, which may conceal potential differences that are specific to the affected side of the body. Furthermore, we prevented the integration of visual information with somatosensory and proprioceptive information. The integration with visual information is an important element under natural situations, which is not addressed in the present study. Additionally, we were not able to compare stimulation of the upper and lower legs because one BIID participant reported that tactile stimulation of the upper alienated leg felt like it was in the desired amputation area. Similarly, we were not able to compare clenching of the left and right hand because one BIID participant had a wish for amputation of his index finger in addition to his leg. Finally, it remains unclear whether altered PM activity is the cause of the feelings of alienation, or whether developmental differences in the feeling of body ownership lead to altered PM processing. Notwithstanding these limitations, our results suggest that feelings of body disownership are associated with reduced PM activity, which may be related to dysfunctional integration of somatosensory and proprioceptive information.
